# Poly-Victimisation among Vietnamese High School Students: Prevalence and Demographic Correlates

**DOI:** 10.1371/journal.pone.0125189

**Published:** 2015-05-01

**Authors:** Minh T. H. Le, Sara Holton, Huong Thanh Nguyen, Rory Wolfe, Jane Fisher

**Affiliations:** 1 Jean Hailes Research Unit, School of Public Health and Preventive Medicine, Monash University, Melbourne, Victoria, Australia; 2 Faculty of Social Sciences, Behaviours and Health Education, Hanoi School of Public Health, Hanoi, Vietnam; 3 Department of Epidemiology and Preventive Medicine, School of Public Health and Preventive Medicine, Monash University, Melbourne, Victoria, Australia; Örebro University, SWEDEN

## Abstract

**Background:**

Exposure to multiple forms of violence, including abuse and crime is termed poly-victimisation. There has been increasing research interest in poly-victimisation among children and adolescents in high income countries. However, experiences among adolescents living in low- and lower-middle-income countries are yet to be examined.

**Aims:**

To establish the prevalence of lifetime exposure to poly-victimisation and demographic characteristics of victims among high school students in Vietnam.

**Methods:**

A cross-sectional survey with a self-report, anonymous questionnaire was conducted in ten high schools in Hanoi, Vietnam between October 2013 and January 2014. Poly-victimisation was assessed using the Juvenile Victimisation Questionnaire Revised 2 (JVQ R2).

**Results:**

A total of 1,606/1,745 (92.0%) eligible students provided data and were included in the analyses. Lifetime exposure to at least one form of victimisation was reported by 94.3% (95%CI: 92.5-95.4%) of participants and lifetime exposure to more than 10 forms by 31.1% (95%CI: 27.8-33.5%). Poly-victimisation was associated with experiencing more adverse life events, having a chronic disease or disability, living with a step-parent, experiencing family life as unhappy, being disciplined at school, and living in a rural area. Poly-victimisation experiences differed among students from the three types of high schools in Vietnam.

**Conclusions:**

These data reveal the prevalence and multi-factorial risks of exposure to poly-victimisation among adolescents in Vietnam. Prevalence rates of different forms of victimisation among Vietnamese students, including those previously un-investigated, were higher than those reported in other settings. Poly-victimisation was also common among them. There were certain subgroups who were more vulnerable to poly-victimisation. Further research to understand the broader aspects of adolescence in Vietnam, including poly-victimisation, is thus recommended. Special attention should be paid to specific subgroups in the prevention of violence against children and adolescents in this setting. Education to raise awareness about poly-victimisation among the community is needed.

## Introduction

Experiences and consequences of interpersonal violence [1] have been of growing research interest in recent decades. To date, most research into interpersonal violence has investigated single forms of violence, including physical, sexual and emotional abuse, and neglect. However, this research has been criticised for ignoring the co-occurrence and inter-relationships among different forms of violence. Higgins and McCabe introduced the term “multi-type maltreatment” in 1998 [2] and suggested that investigations of multiple forms of maltreatment were required to “account for variability in the short- and long-term psychological adjustment of children and adults who had experienced various forms of child abuse and neglect” [3]. Subsequently, Finkelhor et al [4] extended this to the construct of “poly-victimisation” which includes other forms of violence, crime and abuse against children and adolescents, including property damage, physical assault, sexual victimisation, exposure to family or community violence and witnessing of family or community violence as well as childhood maltreatment.

Adolescents, young people aged 10 to 19 years old [5], are particularly vulnerable to violence because of their limited autonomy, dependence on others for care, and emerging maturity [6]. There is substantial evidence of the negative impact that single types of violence have on the physical and mental health of adolescent victims, including increased likelihood of risky behaviours and experiences of suicidal thoughts [7–18].

### Poly-victimisation among adolescents in high income countries

As awareness of multi-type maltreatment and poly-victimisation has increased, research in high income countries about experiences of violence among children and adolescents has been extended from the investigation of single, to multiple forms of violence [2, 19–21]. Lifetime exposure to at least one form of victimisation was recorded to be as low as 22% among Australian young adults [3], to as high as 66% among US children and adolescents [22] and 88% among Spanish college students [23]. While 10% of the US sample experienced more than 10 out of the 34 forms of victimisation assessed by the Juvenile Victimisation Questionnaire (JVQ) [22]; only 5% of the Spanish sample did [23]. Among Australian young adults, 18% reported lifetime experience of exposure to two types of physical, sexual, emotional abuses, neglect or bullying and 14% reported three or more types [3]. However, it is noteworthy that different age groups were examined, including 23–24 years old in the Australian study [3], 2–17 in the US [24] and 14–18 in the Spanish [23]. In these studies, poly-victimisation was assessed using different methods (telephone interviews among the US participants and self-completed questionnaire among the Australian and the Spanish), and different instruments (the JVQ for both the US and the Spanish samples and study specific questions for the Australian). These differences may affect the comparability of the results.

In high income countries poly-victimisation has been shown to have independent detrimental effects on the mental health and adjustment capacity of the victims [21, 25–27] even when controlling for exposure to different single forms of victimisation, including physical assault, property crime, peer or sibling victimisation, child maltreatment, sexual victimisation and witness or indirect victimisation.

### Poly-victimisation among adolescents in low and middle-income countries

Even though 90% of the world’s adolescents live in low and middle income countries, evidence about the prevalence and correlates of poly-victimisation among them is scarce and most is from upper-middle income countries. In a sample of 3,155 12-18-year-old high school students in Shandong province China, 85% of whom resided in a rural area, Dong et al [28] found that two thirds of the students reported at least one form of victimisation in the previous year. Poly-victimisation (which was assessed by the JVQ and was defined in this study as exposure to more than four types) was reported by 17%. In another survey in China using the same instrument, Chan reported similar prevalence estimates of 71% reporting experience of at least one form of victimisation and 14% of poly-victimisation [29]. Compared to the Chinese data, findings from a Malaysian study show a much lower prevalence of 22% of adolescents having experienced at least one form of neglect, physical, emotional or sexual victimisation and 3% experiencing all four [30].However, the use of study-specific questions in this survey compared to a validated measure in the two Chinese studies makes the results from Malaysia and China not directly comparable. Evidence from South Africa suggests higher prevalence of exposure to violence among children and adolescents compared to those reported in other settings. Among 617 South African students aged 12–15 years, Kaminer et al [31] found that 93.1% experienced more than one type of violence and more than 50% experienced four or more types, in the six domains investigated (witnessing of community violence, community victimisation, witnessing of domestic violence, domestic victimisation, sexual abuse and school violence). In these studies [28, 30], poly-victimisation was found to be associated with male gender, younger age, lower socioeconomic status, being an only child, poor parent-child relationship and low quality of school and neighbourhood environment.

### Poly-victimisation among adolescents in Vietnam

Although there are more than 30 million children and adolescents in Vietnam, and they account for more than a third of the nation’s population [32], there is limited evidence about poly-victimisation among them. Most previous studies in Vietnam only investigated specific forms of victimisation. The UNICEF Multi Indicator Cluster Survey 3, investigated mothers aged 15–49 years about their care of their under-five year old children and the children’s health and development. Conducted in fifty low and middle income countries, it found that Vietnam was among the countries in which corporal punishment and psychological and physical abuse of children were the most prevalent [33]. Nguyen et al [18] investigated 2,581 grade 6–12 students in Vietnam and found that 67% reported at least one form and 6% all four forms of neglect, physical, emotional and sexual abuse. Bullying by peers was investigated briefly in a study in which health risk behaviours were the main research focus [34]. Male adolescents who were bullied in the previous month were found to be at increased risk of suicidal thoughts compared to those who were not. Intimate partner violence and severe physical violence by family members and other people were assessed in the Survey Assessment of Vietnamese Youth (SAVY) 1 (2004–05) and 2 (2009–10). These surveys recruited nationally representative samples of adolescents and young adults aged 15–24 years [35]; however, experiences of intimate partner violence were only investigated among married adolescents and young adults– the experience of adolescents who are not married has not yet been investigated. Le et al’s [36, 37] secondary analyses of these data found that 3.7% of the SAVY 2 adolescents had ever experienced injuries due to physical violence by a family member; 7.4% due to physical violence outside the family and nearly 23% of the ever-married adolescents had been verbally, physically or sexually abused by their partner. There was also a significant association between marriage under 18 years of age and increased risk of violence by intimate partners. In all of these studies [18, 34, 35], study-specific questions were used instead of validated measures. Overall, most research about violence against children and adolescents in Vietnam has recruited participants from public schools [18, 34], which are only one of the three types of high school in the country. The experiences of adolescents attending private schools and centres for continuing education have not been investigated. There is no published evidence about Vietnamese adolescents’ experiences of other forms of victimisation such as cyber bullying, dating violence and property victimisation. Poly-victimisation is yet to be investigated in this setting.

The aims of this study were to: 1) examine the prevalence of poly-victimisation among high school students in Vietnam and 2) identify the demographic characteristics which distinguish between adolescent non-victims, victims of up to ten forms and poly-victims (victims of more than ten forms) of violence.

## Methods

### Study design

The study used a cross-sectional survey design, and was conducted between October 2013 and January 2014.

### Setting

Vietnam is classified as a lower middle-income country with a 2013 GDP per capita of USD 1,730 [38]. Most children and adolescents live in rural areas [32].

Hanoi, where this study was conducted, is the capital city of Vietnam with a population of more than 6.8 million people [39]. The city has a total of 29 districts, 12 of which are inner-city and the remainder suburban and rural. One inner-city district and one rural district were purposively selected as study sites.

### Selection of study sites

Upon completion of grade 9, all students in Vietnam sit for the national secondary school graduation exam. The results of this exam are used to determine high school entrance. There are three high school (Grades 10–12) types: public schools, private schools and centres for continuing education. Public high schools require higher entrance marks than private high schools. In contrast to high income countries, students from private schools often have lower levels of academic performance compared to those in public schools [40, 41]. For those who do not meet entry requirements to public high schools and whose families cannot afford tuition fees at private schools, centres for continuing education provide an opportunity to continue formal education. Therefore, students in these different academic institutions may differ from each other in terms of academic capability, household socio-economic status and family composition. Schools of each of these types were purposively selected to represent different sub-populations in each of the chosen districts.

Ten schools were selected: two public high schools, two private high schools and one centre for continuing education from each of the two districts. The average class size of each school varied from 30 to 50 students, with public schools having the largest class size and private schools the smallest. In each school, depending on the class size, four to six classes were selected randomly. All students in the selected classes were invited to participate.

### Inclusion criteria

The inclusion criteria of the study were to be a student aged at least 15 years and attending one of the selected classes.

### Sample size

The sample size for this study was based on the prevalence of physical, emotional, or sexual abuses, or neglect reported in Nguyen et al, 2010 [18]. The required sample size varied from 1,222 to 1,686 depending on the prevalence and 1,686 students was enough to detect a difference of 8% and 10%, respectively, in the prevalence of physical abuse among students attending public schools (47.5%), private schools (55.5%) and centres for continuing education (57.5%) at an alpha level of 0.05, a power of 80%, and presuming a response rate of 90%.

### Data source

Data for the study were collected using an anonymous, self-completed questionnaire of fixed-choice items, including study-specific questions and standardised measures.

#### Socio-demographic information

Study-specific questions were used to assess participants’ socio-demographic characteristics: sex, date of birth, religion, ethnicity, family composition, parental educational attainment, parental occupation, family possession of household assets, self-perception of academic results and academic pressure, experience of being disciplined at school (including being named in the class disciplinary book or during the school assembly; parents being asked to meet the teacher and doing cleaning-up duties) and experience of a chronic disease or disability.

#### Adverse life events

Lifetime experience of adverse life events, including exposure to natural disasters, fire, serious accidents or illnesses of self or close family members, parental imprisonment, parental unemployment and homelessness were assessed using 14 items developed and validated among US adolescents by Turner and Butler [42]. These items have been used in investigation of poly-victimisation among a nationally representative sample of US children and adolescents [22, 25, 43–46].

#### Lifetime experience of poly-victimisation

Lifetime experience of poly-victimisation was assessed using the Juvenile Victimisation Questionnaire Revised 2 (JVQ R2) youth self-report lifetime version [47, 48]. There are a total of 34 questions in the JVQ, which allow investigation of five modules including conventional crimes, child maltreatment, peer and sibling victimisation, sexual victimisation and witnessing of direct or indirect victimisation [4].Compared to the original, the JVQ R2 has several additional items for modules such as family violence, school violence, electronic victimisation and neglect. In this survey, three additional items seeking information about exposure to family violence, neglect and online harassment were used, making a total of 37 items. The JVQ-R2 offers a variety of scoring options, including single-item-level, rescored-item-level, module-level, aggregate-level, and total summary scores [47, 48].

The JVQ was found to have good construct validity and acceptable test-retest reliability (agreement between two administrations ranged from 77%-100% and mean test-retest correlation of 0.63) among US children and adolescents [47]. Its internal consistency was demonstrated in a Cronbach Alpha of 0.80 [47]. There is no published information about the use of the JVQ R2 in Vietnam previously. In this sample, the JVQ-R2 appeared to be suitable for use with a Cronbach Alpha of 0.85.

### Ethics

This research involved the collection of highly sensitive data from minors. It required careful consideration of the ethics of informed consent, voluntary participation, protection of privacy and minimisation of harm. These were addressed in the following ways.

First, in order to inform young people and their parents and to give them time to make a decision about whether or not to participate, information packages which contained a detailed account of the study were distributed to all students of the selected classes and their parents several days before the survey was conducted. Participants and their caregivers were given contact details of the researchers (ML & JF) to address any question they might have about the study before the survey date.

Second, it was clearly explained in the information package that participation was voluntary, and that whether or not they participated would not affect their relationship with the teachers or the way they were treated at school. Students aged at least 18 years could choose not to participate by indicating this and then completing homework during the class time in which the questionnaire was administered, or leaving the questionnaire blank and returning it sealed in the envelope provided to each student with the survey form. Parents of students aged less than 18 years could refuse their child’s participation by completing and returning a form (included in the information package) to the researchers indicating that they did not consent. This method of opting-out is the form of consent with which Vietnamese parents are familiar and it has been widely used in research among children and adolescents in Vietnam [18, 35, 49].

Third, participants’ privacy was protected by collecting no individually identifying information on the questionnaires, which were completed anonymously. All participants were given envelopes and asked to put the questionnaire, whether or not it had been completed, in the envelope and to seal it before submitting it. There was no possibility of re-identification of an individual participant. For students whose parents did not give permission for participation, their privacy was protected by giving them the opportunity to return the withdrawal forms together with their blank questionnaire in the envelope. They were thus not being identified to classmates as non-participants.

Fourth, potential harm was minimised by advising students that they did not have to complete any questions about which they felt uncomfortable, and that they could stop completing the questionnaire at any time if they wished to. Contact details of support services and a free telephone helpline for children and adolescents in Vietnam were provided in the Participant Information package. Students were offered the opportunity to speak in private with the researcher (ML) if they felt distressed or wanted to discuss any matters raised in the questionnaire and where they might receive assistance.

Ethics approval for the conduct of the project was granted by all participating schools and centres, the Human Research Ethics Committee of Monash University (project number CF13/1762-2013000897) and the Institutional Review Board of the Hanoi School of Public Health (Application number 013-148/DD-YTCC).

### Procedure

#### Translation and cultural adaptation

The JVQ-R2 was translated by the first author (ML) and reviewed comprehensively by two independent public health professionals bilingual in English and Vietnamese. The whole questionnaire was then pre-tested among four high school students not attending the study schools. The feasibility of the study, and the comprehensibility and acceptability of the revised questionnaire were tested in a pilot survey among one grade-11 class in a rural public school and another in a rural centre for continuing education. These classes were excluded from the main survey.

#### The main survey

We aimed to recruit about 160 students per school and because class size varied among the selected schools, the number of randomly selected classes in each school ranged from 4 to 6.

On the day of the survey, a questionnaire and an envelope were distributed to each student of the selected classes. Those who did not want to participate or who did not have parental consent were advised to leave the questionnaire blank, and asked to stay in the classroom and prepare for the next academic session quietly. The participants were given instructions on how to complete the questionnaire and filled in the questionnaire within a normal 45-minute class session. This was conducted under the instruction of the research team, without the presence of any teacher or school staff. At the end of the session, all students were asked to put the questionnaire into the envelope provided, seal it and return it to the researchers.

### Data management and analyses

Data was entered using Epidata 3.1 [50]. All data analyses were performed using IBM SPSS 20.0 [51] and Stata 12 [52].

A variable representing socioeconomic status was constructed using principal component analysis of 12 questions about household items [36, 37]. This method was derived from the World Bank method to calculate a household wealth index, which is the widely used method to establish socio-economic status in resource-constrained countries [53]. Three factors were identified (see S1 Appendix). Factor 1 had an Eigenvalue of 3.198, factor 2 1.698 and factor 3 1.039. Factor 1 explained 26.6% of the total variance, factor 2 14.1% and factor 3 8.7%. We decided to use factor 1 alone for subsequent analyses as it explained the most variance among the three factors (more than the sum of the other two factors).Values of this factor were then used to rank participants into quartiles (the poorest, 26–50%, 51–75% and the richest), representing the participant’s corresponding socioeconomic status.

Prevalence of different forms of violence was calculated following the JVQ R2 scoring instructions. For each of the 37 questions in the JVQ R2, a “yes” response was coded as 1 and a “no” response as 0 with a total poly-victimisation score being the sum of all responses, ranging from 0–37. Students were categorised into three groups based on their poly-victimisation scores: “non-victims”(scores of 0),“victims”(scores of 1 to 10) and “poly-victims”(scores > 10). Prevalence of eight aggregated modules including property crime, physical assault, maltreatment, peer or sibling victimisation, sexual victimisation, exposure to family violence, exposure to community violence and witnessing of family violence or community violence was calculated following the standard scoring methods for the JVQ R-2 [48]. One-way ANOVA and chi-square tests were performed to examine associations between socio-demographic factors and poly-victimisation.

Since the number of “non-victims” was small, this category was combined with “victims” and a binary variable of poly-victimisation contrasting “non-victims” and “victims” to “poly-victims” was created. Multiple logistic regressions between demographic variables and this binary poly-victimisation variable were conducted. In these multiple logistic regressions, the “don’t know” category was considered not to be meaningful and was thus treated as missing. All missing data were managed using multiple imputation. Analysis using this method has been shown to provide less biased estimates of associations than the use of complete data only or other methods such as mean imputation [54, 55]. The possible mechanism giving rise to missing data was explored by checking the correlation of missingness of each variable with all other variables in the dataset [55] and this exploratory analysis indicated that it was reasonable to make a missing at random (MAR) assumption. Multiple imputation was performed based on a multivariable normal regression where the school, school type and residential area were regular variables and all other variables in the questionnaire were imputed variables. In this imputation, missing values for each of the 37 items of the JVQ were imputed and subsequently poly-victimisation scores were calculated. Forty datasets were created with imputed data replacing missing values. In each of these datasets, imputed values for categorical variables were rounded up to the nearest round number if they were fractional. Multiple logistic regressions between demographic variables and the binary poly-victimisation variable were then performed on each of these imputed datasets separately and the results combined using Rubin’s Rules [56].

## Results

All ten invited schools agreed to become study sites. A total of 47 classes were selected and 1,745 students were eligible and invited to participate. Of these, 120 students were absent on the day of the survey; nine refused to participate (seven student refusals and two parent refusals); 1,616 completed questionnaires were returned—a response rate of 92.5%. Ten of these had more than two thirds of the questions incomplete and were excluded from imputation and analysis.

The demographic characteristics of the sample are presented in Table 1. Most participants lived in a family with both parents, had more than one sibling, and perceived their family to be happy or very happy. Few were affiliated with a religion.

**Table 1:** Demographic characteristics of 1,606 high school students in Vietnam.

Variable	Total
**Age** (N = 1,535)(Mean ± SD)	16.5 ± 1.0
**Gender** (N = 1,599) (n(%))	
Female	729 (45.6)
Male	870 (54.4)
**Religion** (N = 1,533) (n(%))	
No religion	1048 (68.4)
Buddhism	301 (19.6)
Christianity & others	21 (1.4)
Don’t know	163 (10.6)
**Residence** (N = 1,606) (n(%))	
Urban	817 (50.9)
Rural	789 (49.1)
**Socioeconomic status** (N = 1,526) (n(%))	
Lowest 25%	382 (25.0)
26–50%	380 (24.9)
51–75%	347 (22.7)
Highest 25%	417 (27.3)
**Family composition** (N = 1,596) (n(%))	
Both parents	1409 (88.3)
Only one parent	121 (7.6)
One parent and a stepparent	34 (2.1)
None of parents	32 (2.0)
**Mother’s highest educational attainment** (N = 1,562) (n(%))	
Up to secondary school (grade 9)	398 (25.5)
Completion of high school (grade 12)	721 (46.2)
Don’t know	443 (28.4)
**Father’s highest educational attainment** (N = 1,562) (n(%))	
Up to secondary school (grade 9)	424 (27.1)
Completion of high school (grade 12)	705 (45.1)
Don’t know	433 (27.7)
**Number of siblings** (N = 1,417) (n (%))	
Only child	86 (6.1)
One sibling	767 (54.1)
Two siblings	358 (25.3)
Three siblings	132 (9.3)
Four or more siblings	74 (5.2)
**Perception of family happiness** (N = 1,565) (n(%))	
Happy/ very happy	1399 (89.4)
Unhappy/ Very unhappy	166 (10.6)

### Prevalence of victimisation and poly-victimisation

Prevalence of each item in the 37-item JVQ R2, of the eight aggregated modules and of victimisation and poly-victimisation are presented in Table 2. The median number of victimisation types was 7, IQR (3–12).These prevalence estimates and comparison data from China and the US, using the same measure are presented in Table 3. Overall, all prevalence data among the Vietnamese sample were higher than those reported in China and the US. Chan’s sample of Chinese students were comparable to the sample of this study in age (15–17 years old); about 71% had experienced at least one form of victimisation and 14% were considered poly-victims as they had experienced at least four types [29], while among the Vietnamese adolescents these rates were 94.3% and 74.5%, respectively.

**Table 2:** Percentages of different forms of victimisation among 1,606 high school students in Vietnam (lifetime experience).

Victimisation form	Total (N = 1606)^a^ n	%	95% CI of %
**Conventional Crime**			
Robbery: At any time in your life, did anyone use force to take something away from you that you were carrying or wearing?	205	13.0	11.3–14.7
Personal theft: At any time in your life, did anyone steal something from you and never give it back? Things like a backpack, money, watch, clothing, bike, stereo, or anything else?	676	42.8	40.4–45.3
Vandalism: At any time in your life, did anyone break or ruin any of your things on purpose?	751	47.3	44.8–49.8
Assault with weapon: Sometimes people are attacked with sticks, rocks, guns, knives, or other things that would hurt. At any time in your life, did anyone hit or attack you on purpose with an object or weapon? Somewhere like: at home, at school, at a store, in a car, on the street, or anywhere else?	313	19.8	17.9–21.8
Assault without weapon: At any time in your life, did anyone hit or attack you without using an object or weapon?	778	49.0	46.6–51.5
Attempted assault: At any time in your life, did someone start to attack you, but for some reason, it didn't happen? For example, someone helped you or you got away?	337	21.4	19.4–23.4
Threatened assault: At any time in your life, did someone threaten to hurt you when you thought they might really do it?	636	40.0	37.6–42.4
Kidnapping: When a person is kidnapped, it means they were made to go somewhere, like into a car, by someone who they thought might hurt them. At any time in your life, did anyone try to kidnap you?	24	1.5	0.9–2.1
Bias attack: At any time in your life, have you been hit or attacked because of your skin colour, religion, or where your family comes from? Because of a physical problem you have? Or because someone said you were gay?	28	1.8	1.1–2.4
**Child maltreatment**			
Corporal punishment: At any time in your life, has a grown-up in your life spanked, hit or slapped you on the bottom with their bare hand?	982	62.0	59.6–64.4
Physical abuse by caregiver: Not including spanking on your bottom, at any time in your life, has a grown-up in your life hit, beat, kick, or physically hurt you in any way?	856	54.1	51.7–56.6
Emotional abuse: At any time in your life, did you get scared or feel really bad because grown-ups in your life called you names, said mean things to you, or said they didn't want you?	560	35.2	32.9–37.6
Neglect: When someone is neglected, it means that the grown-ups in their life didn't take care of them the way they should. They might not give them enough food, take them to the doctor when they are sick, or make sure they have a safe place to stay. At any time in your life, were you neglected?	191	12.1	10.5–13.7
Family abduction: Sometimes a family fights over where a child should live. At any time in your life, did a parent take, keep, or hide you to stop you from being with another parent?	62	3.9	3.0–4.9
**Peer and sibling victimisation**			
Gang or group assault: Sometimes groups of kids or gangs attack people. At any time in your life, did a group of kids or a gang hit, jump, or attack you?	273	17.2	15.4–19.1
Peer or sibling assault: At any time in your life, did any kid, even a brother or sister, hit you? Somewhere like: at home, at school, out playing, in a store, or anywhere else?	508	32.2	29.8–34.5
Nonsexual Genital Assault: At any time in your life, did any kids try to hurt your private parts on purpose by hitting or kicking you there?	174	11.0	9.5–12.6
Physical Intimidation by peers: At any time in your life, did any kids, even a brother or sister, pick on you by chasing you or grabbing you or by making you do something you didn't want to do?	333	21.1	19.1–23.1
Relational aggression by peers: At any time in your life, did you get scared or feel really bad because kids were calling you names, saying mean things to you, or saying they didn't want you around?	440	27.8	25.6–30.0
Dating violence: At any time in your life, did a boyfriend or girlfriend or anyone you went on a date with slap or hit you? Experienced dating violence by a boy/girlfriend	210^b^	13.3	11.6–14.9
**Sexual victimisation**			
Sexual assault by known adult: At any time in your life, did a grown-up you know touch your private parts when they shouldn't have or make you touch their private parts? Or did a grown-up you know force you to have sex?	94	5.9	4.8–7.1
Sexual assault by unknown adult: At any time in your life, did a grown-up you did NOT know touch your private parts when they shouldn't have, make you touch their private parts or force you to have sex?	93	5.9	4.7–7.0
Sexual assault by peer/ sibling: Now think about kids your age, like from school, a boyfriend or girlfriend, or even a brother or sister. At any time in your life, did another child or teen make you do sexual things?	43	2.7	1.9–3.5
Forced sex: At any time in your life, did anyone try to force you to have sex; that is, sexual intercourse of any kind, even if it didn't happen?	55	3.5	2.6–4.4
Flashing/ sexual exposure: At any time in your life, did anyone make you look at their private parts by using force or surprise, or by "flashing" you?	223	14.1	12.4–15.8
Verbal sexual harassment: At any time in your life, did anyone hurt your feelings by saying or writing something sexual about you or your body?	183	11.6	10.0–13.2
Statutory rape & sexual misconduct: At any time in your life, did you do sexual things with anyone 18 or older, even things you both wanted?	111	7.1	5.8–8.3
**Witnessing and indirect victimisation**			
Witness to domestic violence: At any time in your life, did you SEE a parent get pushed, slapped, hit, punched, or beat up by another parent, or their boyfriend or girlfriend?	189	11.9	10.3–13.5
Witness to parent assault of sibling: At any time in your life, did you SEE a parent hit, beat, kick, or physically hurt your brothers or sisters, not including a spanking on the bottom?	646	40.7	38.3–43.2
Witness to assault with weapon: At any time in your life, in real life, did you SEE anyone get attacked on purpose WITH a stick, rock, gun, knife, or other thing that would hurt? Somewhere like: at home, at school, at a store, in a car, on the street, or anywhere else?	741	46.9	44.4–49.3
Witness to assault without weapon: At any time in your life, in real life, did you SEE anyone get attacked or hit on purpose WITHOUT using a stick, rock, gun, knife, or something that would hurt?	807	51.1	48.7–53.6
Burglary of family household: At any time in your life, did anyone steal something from your house that belongs to your family or someone you live with? Things like a TV, stereo, car, or anything else?	654	41.6	39.1–44.0
Murder of family member or friend: When a person is murdered, it means someone killed them on purpose. At any time in your life, was anyone close to you murdered, like a friend, neighbour, or someone in your family?	122	7.7	6.4–9.0
Exposure to random shootings, terrorism or riots: At any time in your life, were you in any place in real life where you could see or hear people being shot, bombs going off, or street riots?	95	6.0	4.8–7.2
Exposure to war or ethnic conflict: At any time in your life, were you in the middle of a war where you could hear real fighting with guns or bombs?	56	3.6	2.6–4.5
**Family violence and abuse**			
Parental Displaced aggression: At any time in your life, did one of your parents, because of an argument, break or ruin anything belonging to another parent, punch the wall, or throw something?	465	29.4	27.2–31.7
Other family violence exposure: Now we want to ask you about fights between any grown-ups and teens, not just between your parents. At any time in your life, did any grown-up or teen who lives with you push, hit, or beat up someone else who lives with you, like a parent, brother, grandparent, or other relative?	395	25.3	23.1–27.4
**Internet harassment:** Has anyone ever used the Internet to bother or harass you or to spread mean words or pictures about you?	445	28.3	26.0–30.5
**Aggregated victimisation modules**			
Any physical assault	1240	78.5	76.5–80.5
Any property victimisation	1011	63.9	61.5–66.3
Any maltreatment	1029	64.8	62.4–67.2
Any peer-sibling victimisation	946	60.2	57.8–62.6
Any sexual victimisation	420	26.8	24.6–28.9
Any sexual assault (non-consensual physical contact)	192	12.3	10.7–13.9
Any exposure to family violence	893	57.1	54.6–59.5
Any exposure to community violence	1196	75.9	73.8–78.0
Victimisation categories			
Non victims	79	5.6	4.4–6.9
Victims (1–10 types)	894	63.3	60.7–65.8
Poly-victims	440	31.1	27.8–33.5

^**a**^ Total number of students in each school category may vary across different forms of violence due to missing data.

^b^ The other group consisted of adolescents who either did not have a girl/boyfriend or did not experience dating violence by their partner.

**Table 3:** Percentage of students reporting lifetime experience of different aggregated victimisation modules among 1,606 high school students in Hanoi, Vietnam.

Aggregated module	Current study sample (%)	Chinese sample^a^ ^,^ ^c^ (%)	US sample^b^ ^,^ ^d^ (%)
Internet harassment	28.3		7.9^b^
Any physical assault	78.5		71.1^b^
Any property victimisation	63.9		53.2^b^
Any maltreatment	64.8	14.3^a^	32.1^b^
Any peer-sibling victimisation	60.2	21.0^a^	
Any sexual victimisation	26.8	3.2^a^	27.8^b^
Any sexual assault (non-consensual physical contact)	12.3		11.3^b^
Any exposure to family violence	57.1		34.6^b^
Any exposure to community violence	75.9		
Victimisation categories			
Non victims	5.7	28.6^c^	
Victims of 1–3 forms	19.8	57.4^c^	
Victims of 4–10 forms	45.7	14.0^c^	
Poly-victims of 11–14 forms	17.2		
Poly-victims of 15+ forms	14.6		10.2^d^

^a^ Past year experience of a sample of 3,155 students aged 12–18 recruited from schools in Shandong Province, China [28].

^b^ Lifetime experience of 1,175 adolescents aged 14–17 in a national sample of 4,549 US children and adolescents aged 0–17 [57].

^c^ Lifetime experience of 18,341 students aged 15–17 recruited from schools in Hong Kong and five mainland cities in China [29].

^d^ Lifetime experiences of 417 adolescents aged 15–18 in a national sample of 1,467 US children and adolescents aged 2–17 [45].

When prevalence of separate aggregate modules of victimisation, including property victimisation, maltreatment, peer/ sibling victimisation, sexual victimisation, exposure to family violence, exposure to community violence, and Internet harassment, was compared, the results in this study are still higher than corresponding prevalence estimates reported in previous research conducted in both China and the US [22, 28, 57].

### Distinguishing demographic characteristics of non-victims, victims and poly-victims

Demographic differences among non-victims, victims of one to ten types of victimisation and poly-victims (>10 types) are described in Table 4. Female gender, experiencing more adverse life events, having a chronic disease or disability, living with a step-parent, having more siblings, perceiving the family as unhappy or very unhappy, experiencing studying as a great burden, being dissatisfied with academic results, being punished at school, and rural residence, were associated with an increased likelihood of poly-victimisation. Studying in a centre for continuing education, rather than one of the other school types, was protective.

**Table 4:** Victimisation characteristics of different demographic groups among 1,606 high school students in Vietnam.

Variable	Total sample	Non-victim	Victim of 1–10 types	Poly-victim (>10 types)	p-value
**Age** (Mean ± SD)^a^	16.4±1.0	16.2±0.9	16.4±1.0	16.5±0.9	0.1
**Gender** (n, %)^b^					0.008
Female	653 (100)	24 (3.7)	413 (63.2)	216 (33.1)	
Male	755 (100)	55 (7.3)	478 (63.3)	222 (29.4)	
**Religion** (n(%))^b^					0.07
No religion	931 (100)	56 (6.0)	596 (64.0)	279 (30.0)	
Buddhism	266 (100)	16 (6.0)	154 (57.9)	96 (36.1)	
Christianity or others	18 (100)	0	8 (44.4)	10 (55.6)	
Don’t know	142 (100)	4 (2.8)	97 (68.3)	41 (28.9)	
**Number of adverse life events experienced** (Mean ± SD) ^a^	4.1 ±2.3	1.7 ± 1.6	3.7 ± 1.9	5.6 ± 2.4	<0.001
**Experiences a chronic diseases or disability** (n(%))^b^					<0.001
Yes	161 (100)	1 (0.6)	89 (55.3)	71 (44.1)	
No	1174 (100)	74 (6.3)	760 (64.7)	340 (29.0)	
**Socioeconomic status** (n(%)) ^b^					0.4
Lowest 25%	329 (100)	27 (7.3)	211 (64.1)	94 (28.6)	
26–50%	327 (100)	22 (6.7)	196 (59.9)	109 (33.3)	
51–75%	311 (100)	13 (4.2)	205 (65.9)	93 (29.9)	
Highest 25%	372 (100)	18 (4.8)	232 (62.4)	122 (32.8)	
**Family circumstances** (n(%)) ^b^					<0.001
Both parents	1241 (100)	75 (6.0)	805 (64.9)	361 (29.1)	
Only one parent	108 (100)	3 (2.8)	57 (52.8)	48 (44.4)	
One parent and a stepparent	30 (100)	0 (0.0)	8 (26.7)	22 (73.3)	
None of parents	25 (100)	1 (4.0)	16 (64.0)	8 (32.0)	
**Mother’s highest educational attainment** (n(%))^b^					<0.001
Up to secondary school (grade 9)	359 (100)	10 (2.8)	208 (57.9)	141 (39.3)	
Completion of high school (grade 12)	630 (100)	36 (5.7)	406 (64.4)	188 (29.8)	
Don’t know	390 (100)	31 (8.0)	255 (63.4)	104 (26.7)	
**Father’s highest educational attainment** (n(%))^b^					<0.001
Up to secondary school (grade 9)	374 (100)	16 (4.3)	211 (56.4)	147 (39.3)	
Completion of high school (grade 12)	627 (100)	33 (5.3)	412 (65.7)	182 (29.0)	
Don’t know	378 (100)	30 (7.9)	249 (65.9)	99 (26.2)	
**Parental alcohol abuse**					0.001
Yes	262 (100)	7 (2.7)	150 (57.2)	105 (40.1)	
No	1140 (100)	70 (6.1)	740 (64.9)	330 (28.9)	
**Parental drug abuse**					0.3
Yes	18 (100)	0 (0)	10 (55.6)	8 (44.4)	
No	1378 (100)	77 (5.6)	876 (63.6)	425 (30.8)	
**Number of siblings** (Mean ± SD)^a^	1.6±1.3	1.2±0.8	1.6±1.2	1.7±1.6	0.005
**Perception of family happiness** (n(%))^b^					<0.001
Happy/ Very happy	1233 (100)	73 (5.9)	823 (66.8)	337 (27.3)	
Unhappy/ Very unhappy	149 (100)	2 (1.3)	54 (36.2)	93 (62.4)	
**Perception of academic pressure** (n(%))^b^					<0.001
A lot	124 (100)	3 (2.4)	79 (63.7)	42 (33.9)	
Moderate	445 (100)	13 (2.9)	254 (57.1)	178 (40.0)	
A little	781 (100)	57 (7.3)	524 (67.1)	200 (25.6)	
None	61 (100)	5 (8.2)	37 (60.7)	19 (31.2)	
**Academic satisfaction** (n(%))^b^					<0.001
Satisfied/ very satisfied	546 (100)	44 (8.1)	357 (65.4)	145 (26.6)	
Dissatisfied/ very dissatisfied	862 (100)	33 (3.8)	535 (62.1)	294 (34.1)	
**Being punished at school** (n(%))^b^					<0.001
Frequent	67 (100)	5 (7.5)	27 (40.3)	35 (52.2)	
Sometimes	556 (100)	20 (3.6)	326 (58.6)	210 (37.8)	
Rarely	666 (100)	35 (5.3)	450 (67.6)	181 (27.2)	
Never	121 (100)	18 (14.9)	90 (74.4)	13 (10.7)	
**School type** (n(%))^b^					<0.001
Public	612 (100)	15 (2.5)	389 (63.6)	208 (34.0)	
Private	541 (100)	34 (6.3)	333 (61.6)	174 (32.2)	
Centre for continuing education	260 (100)	30 (11.5)	172 (66.2)	58 (22.3)	
**Residence** (n(%))^b^					0.02
Urban	725 (100)	46 (6.3)	477 (65.8)	202 (27.9)	
Rural	688 (100)	33 (4.8)	417 (60.6)	238 (34.6)	

^a^ p-value for one-way ANOVA test

^b^ p-value for chi-square test

Exposure to more adverse life events, the presence of a chronic disease or disability, living with a step-parent, perception of family as unhappy, punishment at school and rural residence increased the risk of poly-victimisation when controlling for other variables in this sample (Table 5). Students who studied in centres for continuing education had lower risk of poly-victimisation compared to those in public schools.

**Table 5:** Multiple logistic regressions between demographic variables and poly-victimisation among a sample of 1,606 high school students in Vietnam.

Variables	Adjusted OR	95% Confidence Interval		P-value
**Individual factors**				
**Age**	1.04	0.90	1.20	0.6
**Gender** (female vs male)	1.11	0.85	1.46	0.4
**Religion** (ref: no religion)				
Others	1.84	0.25	13.46	0.5
Christianity	3.33	1.00	11.11	0.05
Buddhism	1.17	0.84	1.61	0.4
**Number of adverse life events experienced**	**1.51**	**1.41**	**1.61**	**<0.001**
**Presence of chronic diseases** (yes vs no)	**1.74**	**1.19**	**2.55**	**0.004**
**Familial factors**				
**Socio-economic status** (ref: highest 25%)				
Lowest 25%	0.94	0.59	1.50	0.8
26–50%	0.82	0.53	1.28	0.4
51–75%	0.93	0.62	1.39	0.7
**Number of sibling**	1.03	0.93	1.13	0.6
**Who currently lived with** (ref: both parents)				
None of the parents	1.32	0.55	3.18	0.5
Mother/Father & a step-parent	**3.20**	**1.25**	**8.18**	**0.015**
Single parent	1.26	0.78	2.02	0.3
**Mother's education attainment** (up to secondary school vs completion of high school (grade 12))	1.13	0.80	1.61	0.4
**Father's education attainment** (up to secondary school vs completion of high school (grade 12))	1.26	0.89	1.80	0.2
**Parental alcohol abuse** (yes vs no)	0.93	0.67	1.29	0.7
**Parental drug use** (yes vs no)	1.24	0.47	3.29	0.7
**Perceived family happiness** (unhappy/ very unhappy vs happy/ very happy)	**3.46**	**2.28**	**5.26**	**<0.001**
**Academic environment**				
**Perceived academic pressure** (ref: none)				
A lot	0.60	0.29	1.25	0.2
Moderate	1.21	0.65	2.27	0.5
A little	0.75	0.41	1.37	0.3
**Satisfaction with academic results in previous semester** (satisfied/ very satisfied vs dissatisfied/ very dissatisfied)	1.09	0.84	1.43	0.5
**Being punished at school** (ref: never)				
Frequently	**7.51**	**3.31**	**17.05**	**<0.001**
Sometimes	**3.56**	**1.90**	**6.68**	**<0.001**
Rarely	**2.12**	**1.14**	**3.94**	**0.02**
**School type** (ref: public school)				
Centre for continuing education	**0.56**	**0.38**	**0.83**	**0.004**
Private	0.78	0.58	1.05	0.1
**Community factors**				
**Residential area** (Rural vs urban)	**1.59**	**1.21**	**2.09**	**0.001**

## Discussion

This is the first study in Vietnam to investigate poly-victimisation among adolescents systematically and comprehensively. It has a number of strengths. The sample was recruited from the three major types of academic institutions at high school level in Vietnam. The experiences of students in private schools and centres for continuing education were assessed for the first time in this study. The inclusion of schools and centres in both rural and urban areas allowed examination of potential differences between these two settings. Like other studies conducted in Vietnam [18, 34] or China [28, 29] a very high response rate was achieved. Social and cultural factors which include deferential behaviours, respect for academics and few opportunities to participate in research (thus greater interest in participation) might have accounted for this study’s high response rate. Some forms of victimisation which have not been investigated before, including exposure to property victimisation, dating violence and Internet harassment, were examined. The questionnaire underwent a rigorous review process and pilot testing with adolescents in the target group ensuring appropriateness of the language and acceptability of the questionnaire for use among Vietnamese adolescents. A large sample size was obtained, which allowed us to acquire high power in statistical analyses.

Overall, several main important findings were revealed from these data. First, we found that poly-victimisation is highly prevalent among high school students in Vietnam. Second, we were able to show that some previously un-investigated forms of victimisation were also common among them. Third, there were subgroups of students who were more vulnerable to poly-victimisation than others. These results thus contribute significantly to the knowledge about exposure to multiple forms of violence and poly-victimisation among adolescents in Vietnam.

### Prevalence of poly-victimisation in Vietnam and other countries

Victimisation was widespread in this sample of high school students with nearly a third having experienced more than ten forms of victimisation. Comparison figures on poly-victimisation are only available from upper-middle and high income countries and there are none from other low and lower-middle income countries.

There were much higher rates of lifetime victimisation among these Vietnamese adolescents than among secondary school students from China [29] and South Africa [31],which are upper-middle income countries. Compared to China—a country which shares many social and cultural similarities with Vietnam, the prevalence was double that reported by Chan [29]. Poly-victimisation among these Vietnamese adolescents was also more common than those living in South Africa [31].

The same conclusion can be made when the results are compared with those reported from high income countries. The prevalence of poly-victimisation in this sample (31%) is much higher than that reported among Australian 23-24-year-old young adults (14%) [3] and triple that reported by Turner et al (10%) among a national sample of American children and adolescents [22, 45].

As reported in the results, there are also large discrepancies between the prevalence of separate aggregate modules of victimisation in this study in comparison with those reported in China [28] and the US [22, 27]. Although victimisation was assessed using the JVQ in all of these studies, different survey methods and time frames for victimisation experience may have partly contributed to the different prevalence estimates reported. In this study, information about lifetime experience may have resulted in a higher prevalence compared to those reported among Dong et al’s sample of Chinese students about previous year experience [28]. The use of anonymous self-completed surveys may have overcome the constraints of interviews, which were used in surveys among the US children and adolescents [45, 57], resulting in higher prevalence in this study. However, in comparison with surveys among Chinese students [29] in which the same instrument, survey method and timeframe for victimisation experience were used; prevalence in this sample was still much higher. This suggests that there is a higher risk of lifetime exposure to multiple forms of victimisation among Vietnamese adolescents, compared to those in China.

There are several potential explanations for these results. First, despite increasing awareness among Vietnamese people of the need for child protection, application of harsh corporal punishment in child discipline is common and considered acceptable among a large proportion of the Vietnamese population. Children and adolescents may be frequently verbally or physically maltreated by parents, adult caregivers and teachers at school. Second, students may not be aware of the potential harmful effects bullying may have on their friends and/ or siblings (they thus consider bullying normal and even do it on purpose frequently).Third, the close communal lifestyle, especially in rural areas, may render witnessing of fights and arguments in the villages to be common among children and adolescents.

### Comparison with prior evidence about prevalence of child maltreatment from Vietnam

The findings are similar to prior findings about the high prevalence of corporal punishment of children [58] and the high prevalence of physical, emotional and sexual abuse and neglect of adolescents in Vietnam [18]. However, the prevalence of physical and sexual abuse observed in this sample was higher than those reported in Nguyen et al’s study [18] while the prevalence of neglect was lower. Nguyen et al used study-specific questions rather than standardised measures to assess violence. In addition, response options for each of their questions about violence ranged from never (0), rarely (1), sometimes (2) to frequently (3). A mean score for all participants for each type of violence was calculated and prevalence was defined based on the proportion of students with a score higher than the mean score. Therefore, in addition to differences in participants’ age, instruments used to assess violence, and inclusion of private schools and centres for continuing education in our study, the use of mean values as the cut-off points to define “abuse” in Nguyen et al’s study is likely to explain the differences in findings.

### Prevalence of previously un-investigated forms of victimisation

These data also contribute to understanding the prevalence of property victimisation, physical dating violence and the newly-emerging form of victimisation—Internet harassment, among high school students in Vietnam. Nearly two thirds of the sample had ever had their property deliberately ruined, broken or stolen and more than 28% had ever been harassed on the Internet. There had been no published data from Vietnam about these forms of victimisation to allow comparison with these prevalence estimates. Compared to those reported among children and adolescents in the US [57], Vietnamese high school students had more than three times increased risk of being harassed online, despite the proportion of Internet users per population in Vietnam only half that in the US [59]. It is noteworthy that there was a surge in the proportion of Internet users in Vietnam from 0.25% of the population in 2000 to 43.9% in 2013 [59]. The popularity and wide usage of the Internet, especially social networking sites such as Facebook, among adolescents in Vietnam without proper supervision and appropriate education may make them subjected to increased risk of being victimised online.

The results also reveal that more than a fifth of the sample experienced physical violence perpetrated by their boy/girlfriend. Physical dating violence reported in this sample is five times higher than that reported by Le et al [37] among married 14–25 year-old Vietnamese adolescents. This suggests that intimate partner violence may also be prevalent among adolescents who are unmarried, but in a relationship. Examination of intimate partner violence that is restricted to married adolescents and young adults, therefore, will provide an underestimate of the magnitude of the problem.

### Most vulnerable groups had distinct individual, familial and community characteristics

This study found statistically significant differences in demographic characteristics between non-victims, victims of one to ten forms of victimisation and poly-victims. These characteristics not only pertain to those at the individual level (experience of adverse life events; experience of a chronic disease or disability), but also the familial level, including family composition (the presence of a step-parent), and the community level, such as school type and rural or urban residence.

#### At the individual level

We found no significant association between boys and girls with regards to the risk of poly-victimisation. This finding is contrary to that observed in Dong et al’s [28] sample of Chinese students aged 12–18 years. Male students in Dong et al’s sample who resided in rural areas were more likely to experience multiple forms of victimisation than their female peers. Despite the similarity in terms of cultures between the two nations, social differences such as economic development, educational attainment and social policy (the one-child policy in China versus the one to two children per family in Vietnam) might have contributed to these contradictory findings. It may be that in China, higher expectation towards boys makes them more vulnerable to be poly-victimised. Another explanation would be the different cut-off points used to determine “poly-victims” in this study and Dong et al’s, which were ten and four, respectively.

#### At the familial level

The presence of a step-parent was an important correlate of poly-victimisation in this sample, even when controlling for other variables. In these families, conflicts between the step-parent, the step-children and the child might contribute to the child’s higher risk of being victimised. This finding is consistent with evidence from another Vietnamese study [60]. Nguyen found that Vietnamese adolescents living in families where parents were divorced or where there was a presence of a step-mother were at higher risks of emotional abuse and neglect. Higher levels of parent-child attachment among families with both parents may have been protective of adolescents against being victimised, compared to families of single-parent or with a step-parent.

In this sample, there was also a significant association between adolescents’ risk of being poly-victimised and their perception of family happiness, which was observed in previous research [60]. However, it is not possible to ascertain the direction of this relationship using cross-sectional data. Adolescents living in families in which relationships are poor might be more likely to be poly-victimised or those who are victimised might be more likely to perceive family relationships as poor. It is also noteworthy that the respondents might have had different opinions as to what constituted a happy family.

The type of academic environment appears to play an important role in adolescents’ risk of being poly-victimised. Those who reported high frequency of being punished at school were more likely to be poly-victimised. There were also significant differences among students from public schools, private schools and centres for continuing education with regards to their risk of poly-victimisation. Students in centres for continuing education are often more likely to come from families of lower socio-economic status and have poorer academic performance. Teachers from these centres may therefore pay more attention to the students, in terms of academic, personal and familial aspects. A stronger student-teacher relationship and student-school connectedness may have been built, in comparison with those from public and private schools. These may thus act as protective factors for students in these centres against being victimised. These differences highlight the importance of inclusion of students from different school types in school-based research in Vietnam.

#### At the community level

At the community level, urban-rural residence was a significant correlate of poly-victimisation in this sample. Adolescents who came from rural areas were more likely to report exposure to poly-victimisation compared to their urban counterparts. This could be attributed to a number of factors which may include advancement in economics, advancement in education and public awareness in urban areas about the detrimental impacts of violence against children and adolescents. Urban residents thus benefit from decreased risk of being poly-victimised. It is noteworthy that Vietnam has more than 1,000 years under the rule of various Chinese emperors; Chinese ideology including the Confucians, in which children are expected to be highly disciplined, has been rooted deeply in Vietnamese society. While in urban areas, rapid development and globalisation bring modern ideology to child discipline and parenting as well as the need for child protection, this may not be the case in rural areas. Rural adolescents were also found to be more likely to be sexually abused than urban teenagers, but not for physical, emotional abuse and neglect [60].

### Study limitations

We acknowledge several limitations in this study. First, although schools were located in diverse areas of both rural and urban districts and none of the schools or centres we approached refused to participate, the schools were not randomly selected. Out-of-school adolescents were not included; the sample may thus not be representative of Vietnamese adolescents in general. Second, the traditional method of back-translation when a scale is applied in countries other than the country where it was developed was not applied for the JVQ R2 in this research. Third, recall bias and shame may have contributed to an underestimate of the true prevalence. Fourth, the nature of a cross-sectional survey prevents conclusions about causal-effect relationships to be made, however, many variables included in the multivariate analyses precede poly-victimisation.

## Conclusion

This study advances significantly the evidence from low and lower-middle-income countries about exposure to poly-victimisation among adolescents. The data revealed a high prevalence of exposure to different forms of victimisation and poly-victimisation among high school students in Vietnam. There are certain groups who are more vulnerable to poly-victimisation. These results have important implications for research, education and policy in Vietnam. In terms of research, future comprehensive investigations which include multiple forms of violence, rather than single forms, should be conducted. Inclusion of both married and unmarried, but partnered people in investigation of intimate partner violence in Vietnam is recommended. The role of individual, familial and community factors in adolescents’ risk of being poly-victimised should be investigated further in longitudinal research. Experience of adolescents attending different types of schools may differ; experience of out-of-school adolescents remained un-investigated; inclusion of students from different school types as well as out-of-school adolescents is thus needed.

Despite the Law on Care, Protection and Education of Children being implemented in Vietnam in 2004, it appears not to have been effective. According to this law, child maltreatment and violence against children are illegal. However, there has not been mandatory reporting of these actions in Vietnam and many children and adolescents are still being abused or victimised.

It is suggested that education is needed to raise public awareness about violence against children and adolescents in Vietnam. Comprehensive intervention programs which aim to prevent violence in the family, school and community should be established. Enforcement of Child protection policy in Vietnam should be considered with more attention to the most vulnerable groups. More involvement of not only policy makers, child protection authorities, but also families, schools and communities is essential in prevention of violence against children and adolescents in this country.

## Supporting Information

S1 Appendix Results from principal component analysis of 12 questions about possession of household items. (DOCX)
