# Supplementary material for: Poly-Victimisation among Vietnamese High School Students: Prevalence and Demographic Correlates
Source: PLoS One. 2015 May 1;10(5):e0125189. doi: 10.1371/journal.pone.0125189 (PMC4416888; doi:10.1371/journal.pone.0125189)
Supplement: S1 Appendix — (DOCX) [file pone.0125189.s001.docx]

# Appendix

**Appendix 1: Results from principal component analysis of 12 questions about possession of household items**

| **Factor loadings^a^** | | | |
| --- | --- | --- | --- |
|  | Factor | | |
|  | 1 | 2 | 3 |
| Internet | 0.740 | -0.329 | 0.000 |
| Computer | 0.724 | -0.283 | -0.019 |
| Air condition | 0.682 | -0.262 | -0.055 |
| Washing machine | 0.647 | 0.016 | -0.082 |
| Refrigerator | 0.481 | 0.546 | -0.102 |
| Motorbike | 0.463 | 0.494 | -0.186 |
| CD, DVD or VCD | 0.459 | 0.031 | -0.082 |
| Home phone | 0.438 | -0.266 | 0.433 |
| Car | 0.423 | -0.358 | 0.063 |
| Mobile phone | 0.340 | 0.534 | 0.004 |
| Television | 0.318 | 0.604 | 0.123 |
| Bicycle | 0.023 | 0.220 | 0.878 |
| Extraction Method: Principal Component Analysis. | | | |
| ^a^ 3 components extracted. | | | |
